# Second to fourth digit ratio (2D:4D) and prostate cancer risk in the Melbourne Collaborative Cohort Study

**DOI:** 10.1038/bjc.2011.253

**Published:** 2011-07-05

**Authors:** D C Muller, G G Giles, J T Manning, J L Hopper, D R English, G Severi

**Affiliations:** 1 Cancer Epidemiology Centre, Cancer Council of Victoria, Melbourne, Victoria, Australia; 2 Centre for Molecular, Environmental, Genetic, and Analytic Epidemiology, The University of Melbourne, Melbourne, Victoria, Australia; 3 MRC Epidemiology Research Centre, University of Southampton, Southampton General Hospital, Southampton, UK

**Keywords:** digit ratio, 2D:4D, prostate cancer, cohort study

## Abstract

**Background:**

The ratio of the lengths of index and ring fingers (2D:4D) is a marker of prenatal exposure to sex hormones, with low 2D:4D being indicative of high prenatal androgen action. Recent studies have reported a strong association between 2D:4D and risk of prostate cancer.

**Methods:**

A total of 6258 men participating in the Melbourne Collaborative Cohort Study had 2D:4D assessed. Of these men, we identified 686 incident prostate cancer cases. Hazard ratios (HRs) and confidence intervals (CIs) were estimated for a standard deviation increase in 2D:4D.

**Results:**

No association was observed between 2D:4D and prostate cancer risk overall (HRs 1.00; 95% CIs, 0.92–1.08 for right, 0.93–1.08 for left). We observed a weak inverse association between 2D:4D and risk of prostate cancer for age <60, however 95% CIs included unity for all observed ages.

**Conclusion:**

Our results are not consistent with an association between 2D:4D and overall prostate cancer risk, but we cannot exclude a weak inverse association between 2D:4D and early onset prostate cancer risk.

The ratio of the lengths of the index (2D) and ring (4D) fingers as measured by the ratio 2D:4D has been suggested as a proxy indicator of prenatal androgen activity, with low 2D:4D reflecting higher *in utero* testosterone exposure (Manning *et al*, 1998; McIntyre, 2006; Hönekopp and Watson, 2010). There are several lines of evidence indicating that 2D:4D is affected by prenatal androgens (Breedlove, 2010), and that digit ratios are longitudinally stable (McIntyre *et al*, 2005; Trivers *et al*, 2006). Hormone exposure in early life has been implicated in the aetiology of numerous cancers (Potischman *et al*, 2005). Prostate cancer is a hormonally driven and a regulated disease, but studies have failed to detect associations between a single measure of hormone levels in adulthood and prostate cancer risk (Roddam *et al*, 2008).

Two recent studies have aimed to assess whether 2D:4D is associated with prostate cancer (Jung *et al*, 2011; Rahman *et al*, 2011). Both of these studies concluded that low 2D:4D, and thereby high prenatal testosterone, is a marker of increased risk of prostate cancer. We examine whether 2D:4D is associated with prostate cancer risk in a large sample of men participating in the Melbourne Collaborative Cohort Study (MCCS).

## Materials and methods

The MCCS is a prospective cohort study of 41 514 people (17 045 men) recruited between 1990 and 1994, 99.3% of whom were aged 40–69 years. Details of the MCCS have been published previously (Giles and English, 2002). At a recent face-to-face follow-up conducted during 2003–2009, 6287 men had their hands photocopied. The length of the index and ring fingers were measured from photocopies of the surface of the hand using digital Vernier calipers. The length of the index finger was divided by the length of the ring finger to obtain 2D:4D, and Dr-l was defined as the difference between right and left 2D:4D. Measurement was undertaken by a team of trained research assistants at Cancer Council Victoria.

Follow-up commenced at baseline attendance and ended at 30 June 2009, the date the participant left Australia, diagnosis of an unknown primary tumour, or death, whichever occurred first. Of 6287 men who attended follow-up, we excluded 29 with a pre-baseline diagnosis of invasive prostate cancer or unknown primary tumour, leaving 6258 men available for analysis. During a median follow-up of 16 years, 686 incident prostate cancer cases were identified via linkage to the Victorian Cancer Registry.

### Statistical analysis

Overall hazard ratios (HRs) and 95% confidence intervals (CIs) for a standard deviation (s.d.) increase in 2D:4D measures were obtained from Weibull models with age as the time axis. Age-varying HRs were estimated by flexible parametric survival models (Lambert and Royston, 2009), incorporating restricted cubic splines with six knots to model the baseline hazard, and restricted cubic splines with one knot to allow the HR to vary with age. Knots were evenly spaced across the distribution of uncensored log survival times. Separate estimates of HRs and CIs for age <60 and ⩾60 years were obtained by fitting Weibull models to time-split data. The data duplication method was used to fit competing risks Weibull models for tumour aggressiveness (Lunn and McNeil, 1995). Aggressive tumours were those with Gleason score >7 or stage IV, or having prostate cancer as cause of death. Separate models were fit for right and left 2D:4D and Dr-l, and all models were adjusted for country of birth and baseline smoking status, as these factors are associated with 2D:4D (Manning *et al*, 2000; Manning and Fink, 2011). Statistical analyses were performed using Stata 11.1 for Linux (Stata Corporation, College Station, TX, USA).

## Results

Characteristics of the study population are shown in Table 1. Non-cases were younger than cases on average, with median age at baseline of 54 years compared with 59 years. Seventy-one percent of participants were born in Australia, New Zealand, or in the United Kingdom.

**Figure 1:**
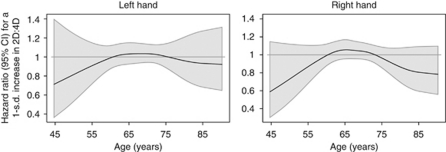
Time-varying HRs and 95% CIs from flexible parametric time to event models, adjusted for ethnicity and baseline smoking status.

Estimates from Weibull models are presented in Table 2. We found no overall association between either left or right 2D:4D and prostate cancer risk (HRs 1.00; 95% CIs, 0.92–1.08 for right, 0.93–1.08 for left). Estimated age-varying HRs and 95% CIs are plotted in Figure 1. There is some indication that higher 2D:4D is associated with lower early onset prostate cancer risk. For instance, the HR for an increase of one standard deviation in 2D:4D and prostate cancer risk at an age of 55 years is ≈0.80 (95% CI, ≈0.65–1.10) for both hands. Similarly, risk of prostate cancer for men older than 80 years appears to be slightly reduced with higher 2D:4D; however, CIs include unity for all observed ages. Splitting follow-up at an age of 60 years and estimating separate HRs for the two age brackets also suggests that higher 2D:4D might be associated with decreased prostate cancer risk before 60 years of age. The HRs for an increase of one standard deviation in 2D:4D and risk of prostate cancer for age <60 years were 0.91 (95% CI, 0.73–1.13) and 0.88 (95% CI, 0.71–1.10) for left and right hands, respectively. Estimated HRs did not vary substantially by tumour aggressiveness (Table 2). No associations were observed for Dr-l (data not shown).

## Discussion

We found no statistically discernable association between 2D:4D and risk of prostate cancer either overall or by age; however, we cannot exclude that there might be a small inverse association between 2D:4D and risk of prostate cancer diagnosed before the age of 60 years.

Two recent studies have examined whether 2D:4D is associated with prostate cancer risk. The first was a clinical cohort study of 366 Korean men presenting with lower urinary tract symptoms (Jung *et al*, 2011). This study found that the odds of being diagnosed with prostate cancer were significantly higher for men with low 2D:4D compared with high 2D:4D (OR 3.22, 95% CI, 1.33–7.78). The second was a large case–control study, which reported an inverse association between self-assessed right 2D:4D and odds of prostate cancer (OR for index finger longer than ring finger *vs* index finger shorter than ring finger 0.67, 95% CI, 0.57–0.80) (Rahman *et al*, 2011). This study also reported a remarkably strong association between 2D:4D and prostate cancer diagnosed before the age of 60 years (OR 0.13, 95% CI, 0.09–0.21).

Although we found no evidence for an association of the magnitude previously reported (and indeed no statistically discernable associations at all), the possibility that there might be a weak inverse association between 2D:4D and early onset prostate cancer risk is broadly consistent with the estimate reported by Rahman *et al* (2011), as well as the current understanding of the association between hormone exposure *in utero* and 2D:4D. There are several differences between the studies that could account for the differing results, from study design (population based cohort *vs* clinical cohort *vs* case–control), to digit measurement method (photocopy *vs* direct *vs* self-reported assessment).

Our study has the advantage of being a large, population-based cohort with complete follow-up in terms of cancer diagnosis. Digit measurements were made with a high degree of reliability by trained research assistants. A disadvantage of our study is that not all participants attended follow-up, and thus we do not have 2D:4D available for every participant. If 2D:4D is associated with disease severity, and if disease severity is in turn associated with attendance, estimated associations could be biased. As there is evidence that 2D:4D is stable over time (McIntyre *et al*, 2006; Trivers *et al*, 2006), we consider it appropriate to analyse these data prospectively, despite the retrospective collection of 2D:4D. Another limitation of our study is the relatively small number of early onset prostate cancer cases, leading to limited power to detect associations between 2D:4D and prostate cancer risk for younger men.

Our analysis does not confirm the strong inverse association between 2D:4D and risk of prostate cancer previously reported. We cannot exclude the possibility that high 2D:4D is associated with lower risk of early onset prostate cancer. High 2D:4D is a marker of low *in utero* testosterone exposure, and thus hormone activity, early in development, might impact upon later risk of prostate cancer. Further research is required to clarify any association between 2D:4D and prostate cancer, especially for younger men.

## Figures and Tables

**Table 1:** Characteristics of the 6258 men by prostate cancer case status

	**Cases (*n*=686)**	**Controls (*n*=5572)**	**Total (*n*=6258)**
Baseline age (median, inter-quartile range)	59 (54–64)	54 (47–61)	55 (47–62)
Right 2D:4D (mean, s.d.)	0.944 (0.039)	0.947 (0.037)	0.947 (0.037)
Left 2D:4D (mean, s.d.)	0.952 (0.036)	0.954 (0.037)	0.953 (0.036)
			
*Baseline smoking status*^a^, n *(%)*			
Never smoked	309 (45)	2432 (44)	2741 (44)
Current smoker	56 (8)	715 (13)	771 (12)
Former smoker	321(47)	2423 (43)	2744 (44)
			
*Country of birth*, n *(%)*			
Australia, New Zealand, United Kingdom	571 (83)	3875 (70)	4446 (71)
Italy	81 (12)	1012 (18)	1093 (17)
Greece	34 (5)	685 (12)	719 (12)

Abbreviation: 2D:4D=ratio of the lengths of the index (2D) and ring (4D) fingers.

a Two men were missing information on baseline smoking status.

**Table 2:** HRs and 95% CIs for a one standard deviation increase in 2D:4D and prostate cancer risk overall, by age, and by tumour aggressiveness

	**Left hand**		**Right hand**	
	**HR (95% CI)**	***P*-value**	**HR (95% CI)**	***P*-value**
Overall^a^	1.00 (0.93–1.08)	0.98	1.00 (0.92–1.07)	0.92
				
*By age* b		0.35		0.24
< 60 years	0.91 (0.73–1.13)		0.88 (0.71–1.10)	
⩾60 years	1.02 (0.94–1.11)		1.01 (0.93–1.10)	
				
*Tumour aggressiveness* c		0.07		0.49
Non-aggressive	1.04 (0.96–1.14)		1.01 (0.93–1.10)	
Aggressive	0.88 (0.75–1.04)		0.94 (0.79–1.13)	

Abbreviations: CI=confidence interval; HR=hazard ratio; 2D:4D=ratio of the lengths of the index (2D) and ring (4D) fingers.

a From Weibull model, with age as the time metric, adjusted for ethnicity and baseline smoking status. *P*-values are from likelihood ratio tests of the 2D:4D variables.

b From Weibull model, with age split at 60 years, adjusted for ethnicity and baseline smoking status. *P*-values are from likelihood ratio tests of the interaction between 2D:4D and the split timescale.

c From competing risks Weibull model with age as the time metric, adjusted for ethnicity and baseline smoking status. *P*-values are from likelihood ratio tests of heterogeneity by aggressiveness. Aggressive cases are those with total Gleason score >7 and/or tumour stage IV. Prostate cancer cause-specific deaths have also been included as aggressive cases.

## References

[bib1] Breedlove SM (2010) Minireview: organizational hypothesis: instances of the fingerpost. Endocrinology 151: 4116–4122 20631003 10.1210/en.2010-0041 PMC2940503

[bib2] Giles GG, English DR (2002) The Melbourne Collaborative Cohort Study. IARC Sci Publ 156: 69–70 12484128

[bib3] Hönekopp J, Watson S (2010) Meta-analysis of digit ratio 2D:4D shows greater sex difference in the right hand. Am J Hum Biol 22: 619–630 20737609 10.1002/ajhb.21054

[bib4] Jung H, Kim KH, Yoon SJ, Kim TB (2011) Second to fourth digit ratio: a predictor of prostate-specific antigen level and the presence of prostate cancer. BJU Int 107: 591–596 20633006 10.1111/j.1464-410X.2010.09490.x

[bib5] Lambert PC, Royston P (2009) Further development of flexible parametric models for survival analysis. Stata J 9: 265–290(26)

[bib6] Lunn M, McNeil D (1995) Applying Cox regression to competing risks. Biometrics 51: 524–532 7662841

[bib7] Manning JT, Barley L, Walton J, Lewis-Jones DI, Trivers RL, Singh D, Thornhill R, Rohde P, Bereczkei T, Henzi P, Soler M, Szwed A (2000) The 2nd:4th digit ratio, sexual dimorphism, population differences, and reproductive success. Evidence for sexually antagonistic genes? Evol Hum Behav 21: 163–183 10828555 10.1016/s1090-5138(00)00029-5

[bib8] Manning JT, Fink B (2011) Digit ratio, nicotine and alcohol intake and national rates of smoking and alcohol consumption. Pers Indiv Differ 50: 344–348

[bib9] Manning JT, Scutt D, Wilson J, Lewis-Jones DI (1998) The ratio of 2nd to 4th digit length: a predictor of sperm numbers and concentrations of testosterone, luteinizing hormone and oestrogen. Hum Reprod 13: 3000–3004 9853845 10.1093/humrep/13.11.3000

[bib10] McIntyre MH (2006) The use of digit ratios as markers for perinatal androgen action. Reprod Biol Endocrinol 4: 10 16504142 10.1186/1477-7827-4-10 PMC1409789

[bib11] McIntyre MH, Cohn BA, Ellison PT (2006) Sex dimorphism in digital formulae of children. Am J Phys Anthropol 129: 143–150 16224778 10.1002/ajpa.20240

[bib12] McIntyre MH, Ellison PT, Lieberman DE, Demerath E, Towne B (2005) The development of sex differences in digital formula from infancy in the FELS Longitudinal Study. Proc Biol Sci 272: 1473–1479 16011922 10.1098/rspb.2005.3100 PMC1559827

[bib13] Potischman N, Troisi R, Thadhani R, Hoover RN, Dodd K, Davis WW, Sluss PM, Hsieh C-C, Ballard-Barbash R (2005) Pregnancy hormone concentrations across ethnic groups: implications for later cancer risk. Cancer Epidemiol Biomarkers Prev 14: 1514–1520 15941965 10.1158/1055-9965.EPI-04-0869

[bib14] Rahman AA, Lophatananon A, Stewart-Brown S, Harriss D, Anderson J, Parker T, Easton D, Kote-Jarai Z, Pocock R, Dearnaley D, Guy M, O’Brien L, Wilkinson RA, Hall AL, Sawyer E, Page E, Liu JF, Eeles RA, Muir K (2011) Hand pattern indicates prostate cancer risk. Br J Cancer 104: 175–177 21119657 10.1038/sj.bjc.6605986 PMC3039824

[bib15] Roddam AW, Allen NE, Appleby P, Key TJ (2008) Endogenous sex hormones and prostate cancer: a collaborative analysis of 18 prospective studies. J Natl Cancer Inst 100: 170–183 18230794 10.1093/jnci/djm323 PMC6126902

[bib16] Trivers R, Manning J, Jacobson A (2006) A longitudinal study of digit ratio (2D:4D) and other finger ratios in Jamaican children. Horm Behav 49: 150–156 16040033 10.1016/j.yhbeh.2005.05.023

